# Cephalochromin Effects in Triple-Negative Breast Cancer Cells: Apoptosis Induction and Modulation of Survival Pathways

**DOI:** 10.1021/acs.jnatprod.5c01020

**Published:** 2025-12-04

**Authors:** Isabelle Diccini, Natália Sudan Parducci, Bruna Oliveira de Almeida, Victor Farinella, Patrick Castilho dos Santos, Livia Bassani Lins de Miranda, Sabrina Mendes Botelho, Keli Lima, Jorge Antonio Elias Godoy Carlos, Anali Del Milagro Bernabe Garnique, Marcelo José Pena Ferreira, Leticia Veras Costa-Lotufo, João Agostinho Machado-Neto

**Affiliations:** † Department of Pharmacology, Institute of Biomedical Sciences, 28133 University of São Paulo, São Paulo, CEP 05508-900, Brazil; ‡ Department of Botany, Institute of Biosciences, University of São Paulo, São Paulo, CEP 05508-090, Brazil; § São Carlos Institute of Chemistry, University of São Paulo, São Carlos, CEP 13563-120, Brazil; ∥ Department of Internal Medicine, Faculty of Medicine, University of São Paulo, São Paulo, CEP 05403-000, Brazil

## Abstract

Triple-negative breast cancer (TNBC) is an aggressive subtype characterized by the absence of estrogen, progesterone, and HER2 receptors, limiting treatment options due to the lack of targeted therapies. Survivin (BIRC5), an inhibitor of apoptosis (IAP) protein, is overexpressed in TNBC and contributes to tumor progression, chemoresistance, and poor prognosis. Cephalochromin, a fungal-derived bioactive compound, has demonstrated cytotoxic activity in various cancer models; however, its effects on breast cancer remain unexplored. In this study, we evaluated the antineoplastic potential of cephalochromin in breast cancer cells, focusing on its impact on cell viability, apoptosis, cell cycle regulation, and survivin modulation. Cephalochromin exhibited potent cytotoxic effects in TNBC models, inducing apoptosis, disrupting cell cycle progression, and downregulating survivin expression. Mechanistically, cephalochromin treatment induced PARP1 cleavage and increased the expression of γH2AX, SQSTM1/p62, and LC3BII. Gene expression analysis revealed the broad modulation of key regulators involved in apoptosis, DNA damage response, and macroautophagy. Furthermore, cephalochromin enhanced the cytotoxicity of paclitaxel and doxorubicin, showing additive synergistic interactions. In conclusion, our study provides compelling evidence of cephalochromin’s antineoplastic activity in breast cancer, highlighting its potential to improve treatment outcomes. Further preclinical studies are warranted to validate their therapeutic efficacy and safety.

## Graphical abstract

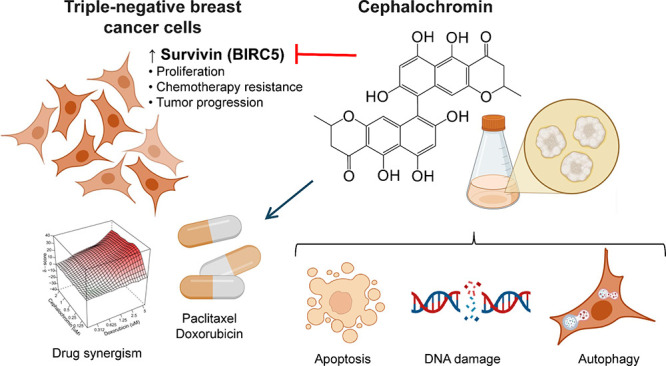

Breast cancer is the most prevalent malignant neoplasm among women and represents one of the leading causes of cancer-related mortality worldwide.^1–3^ Among its various subtypes, triple-negative breast cancer (TNBC) is one of the most aggressive, characterized by the absence of estrogen, progesterone, and HER2 receptors, which limits the available therapeutic options. The treatment of TNBC primarily relies on cytotoxic chemotherapy, which, although initially effective, is often associated with the development of resistance and tumor recurrence.^4–6^ Consequently, there is an urgent need in oncology for novel therapeutic strategies that can overcome these limitations and enhance the efficacy of conventional treatments.

Survivin (also known as BIRC5) is a member of the inhibitor of apoptosis (IAP) protein family that plays a pivotal role in regulating cell division and evading programmed cell death.^7,8^ Its overexpression has been widely implicated in tumor progression, chemotherapy resistance, and poor prognosis in various cancers, including breast cancer.^9,10^ Preclinical studies suggest that targeting survivin may enhance the sensitivity of tumor cells to chemotherapeutic agents, positioning it as a promising target for developing new therapeutic approaches.^11,12^ In breast cancer, survivin is essential for disease progression by regulating the cell cycle, inhibiting apoptosis, and promoting therapeutic resistance. Its overexpression is associated with a more aggressive tumor phenotype, increased cell proliferation, and a diminished response to chemotherapy, contributing to disease progression. Additionally, survivin serves as both a diagnostic and prognostic marker, as its high expression correlates with poorer clinical outcomes, higher recurrence rates, and reduced patient survival. Thus, modulating survivin represents a promising therapeutic strategy to improve the effectiveness of breast cancer treatment.^13,14^

Preliminary research suggests that cephalochromin, a natural bioactive compound derived from fungi (*Cephalosporium sp.*, *Pseudoanguillospora sp.*, and *Verticillium sp*), may modulate molecular pathways associated with autophagy and apoptosis through survivin downregulation, indicating a potential antineoplastic role. The cytotoxic activity of the compound has been previously described for A549 lung cancer cells, although it remains unexplored in the context of breast cancer.^15–17^ Nevertheless, the potential interaction with widely used chemotherapeutic agents in clinical practice remains unclear.

In this context, this study aims to investigate the effects of cephalochromin in breast cancer cell models, evaluating its ability to impact cell viability, apoptosis, and survivin expression. Additionally, we examined whether cephalochromin can potentiate the cytotoxic effects of paclitaxel and doxorubicin, proposing its use as a potential adjuvant agent in breast cancer therapy.

## Results and Discussion

### Cephalochromin Reduces Cell Viability and Clonal Growth of Breast Cancer Cells

We first investigated the effects of cephalochromin on the viability of a panel comprising nonmalignant breast cells and breast cancer cells. Cephalochromin treatment exhibited cytotoxic effects on both malignant and nonmalignant breast cell models, with IC_50_ values ranging from 0.6 to 13.1 μM. The MCF-7 and T47D cell lines, which express hormone receptors for estrogen and progesterone, were the most resistant to the drug (IC_50_: 12.2–13.1 μM). In contrast, TNBC cell lines, particularly MDA-MB-231 (IC_50_: 0.6 μM) and Hs578T (IC_50_: 0.9 μM), were the most sensitive (Figure 1). Furthermore, the selectivity index was favorable (>1) for all TNBC cell lines compared to nonmalignant breast epithelial cells (Table S1). In breast cancer cells, cephalochromin-induced viability reduction was both concentration- and exposure-time-dependent (Figure 1C). Concentration–response curves were also obtained for two clinically used drugs, paclitaxel and doxorubicin, in MDA-MB-321 and Hs578T cells as reference compounds after 72 h of incubation (Figure S1). The IC_50_ obtained values indicated that both compounds were active against both cell lines, nevertheless with moderated cytotoxicity (IC_50_ values for paclitaxel of 0.6 and 7 μM in MDA-MB-321 and Hs578T, respectively; IC_50_ values for doxorubicin of 1.4. and 5.3 μM in MDA-MB-321 and Hs578T, respectively). These values are on the same order of magnitude as the ones obtained with cephalochromin under the same conditions. Additionally, cephalochromin treatment inhibited clonal growth across all breast cancer cell models evaluated (Figure 1D). Interestingly, prolonged drug exposure reduced colony formation in T47D cells but not in MCF-7 cells, even at low concentrations, suggesting that continuous exposure may overcome intrinsic resistance in certain models. In spheroid models of Hs578T and T47D cells, cephalochromin reduced the cell viability in a concentration-dependent manner (Figure S2).

**Figure 1:**
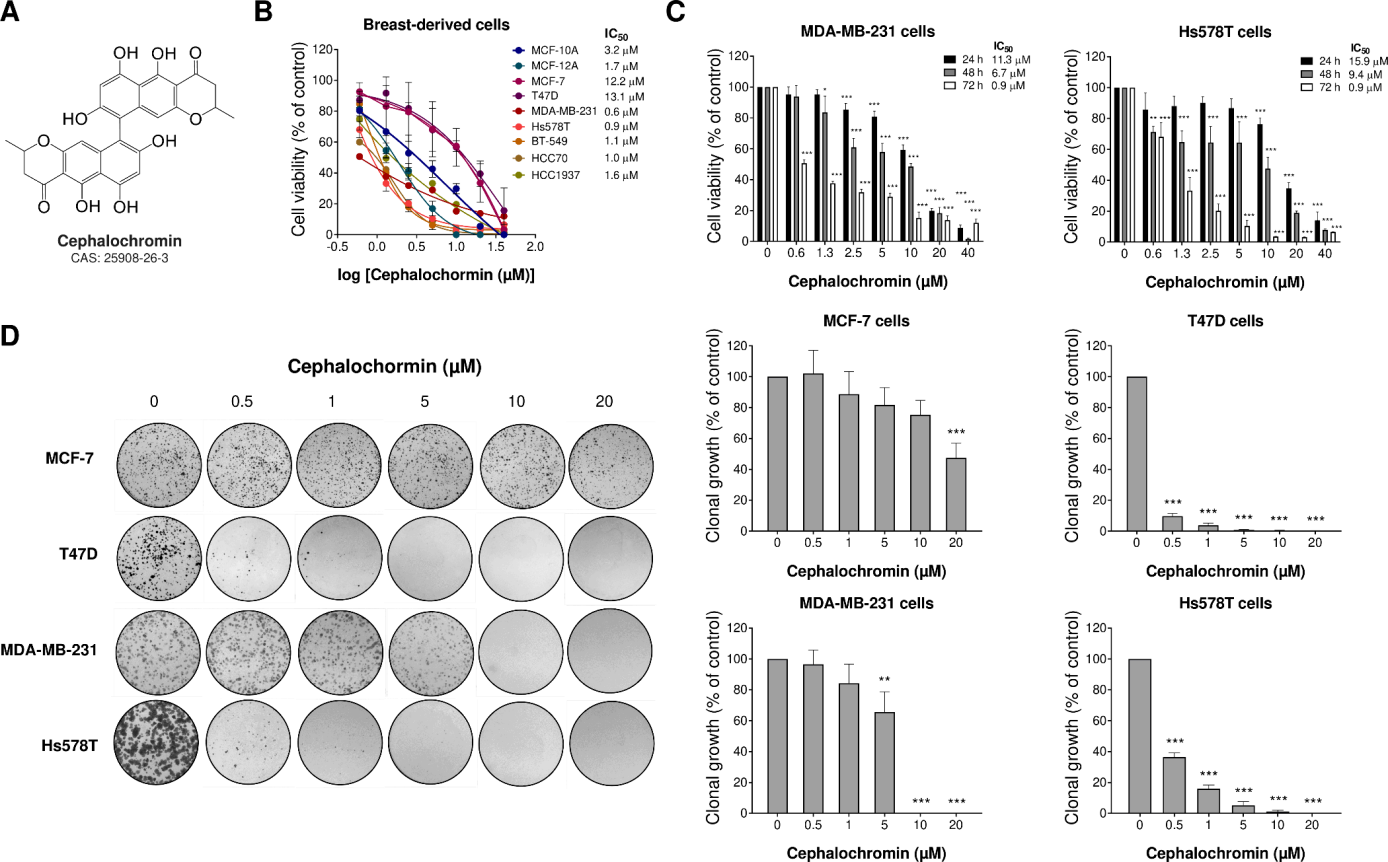
**Cephalochromin suppresses cell viability and clonal expansion in breast cancer cells.** (A) The chemical structure of cephalochromin is shown. (B) Dose-dependent cytotoxicity was evaluated using the methylthiazolyl tetrazolium (MTT) assay in a panel of nonmalignant human breast epithelial cell lines (MCF-10A and MCF-12A) and breast cancer cell lines, including estrogen and progesterone receptor-positive cells (MCF-7 and T47D) and triple-negative cells (MDA-MB-231, Hs578T, BT-549, HCC70, and HCC1937). Cells were treated with either vehicle or increasing concentrations of cephalochromin (0.6–40 μM) for 72 h. (C) Dose- and time-dependent cytotoxicity was assessed using the MTT assay in MDA-MB-231 and Hs578T cells treated with vehicle or increasing concentrations of cephalochromin (0.6–40 μM) for 24, 48, and 72 h. Cell viability was quantified as a percentage relative to that of vehicle-treated controls. Results are expressed as the mean ± SD from at least three independent experiments. IC_50_ values are provided in the figure. (D) Representative images of colony formation assays in breast cancer cells treated with vehicle or cephalochromin (0.5–20 μM) for 10–14 days. The bar graph represents the mean ± SD of the relative number of colonies as a percentage of the control. *p < 0.05, **p < 0.01, ***p < 0.001; ANOVA followed by Bonferroni post hoc test.

A noteworthy finding of our study is the cytotoxic effect of cephalochromin on nonmalignant mammary epithelial cells, despite its favorable selectivity indices against TNBC cells. Using a panel of nonmalignant and malignant breast cell lines, we observed that survivin levels in MCF-12A cells were comparable to those found in several breast cancer cell lines (Figure S3). This suggests that during the immortalization process, certain neoplastic characteristics, such as high survivin expression, may have been incorporated, given that survivin levels are typically very low in normal breast tissues.^13,14^ In this context, cephalochromin may have potential activity in preneoplastic breast lesions and could prevent disease progression.

### Cephalochromin Impairs Cell Cycle Progression and Induces Apoptosis in Breast Cancer Cells

We then selected the most cephalochromin-sensitive TNBC cell lines, MDA-MB-231 and Hs578T, for further investigation. Cell cycle analysis revealed a concentration-dependent increase in subG1 cell populations rather than a reduction in cycling MDA-MB-231 cells following drug exposure, suggesting the induction of DNA damage and apoptosis. In Hs578T cells, a similar concentration-dependent increase in subG1 cells was observed; however, at lower concentrations, an accumulation of cells in the G_2_/M phase was detected, indicating activation of the DNA damage-associated cell cycle checkpoint (Figure 2A). Apoptosis assessment via phosphatidylserine externalization, measured by annexin V labeling, confirmed the induction of concentration-dependent cell death in both models (Figure 2B). Moreover, these findings highlight the greater sensitivity of MDA-MB-231 cells to apoptosis, further corroborating the increase in sub-G1 cell populations observed in the cell cycle analysis.

**Figure 2:**
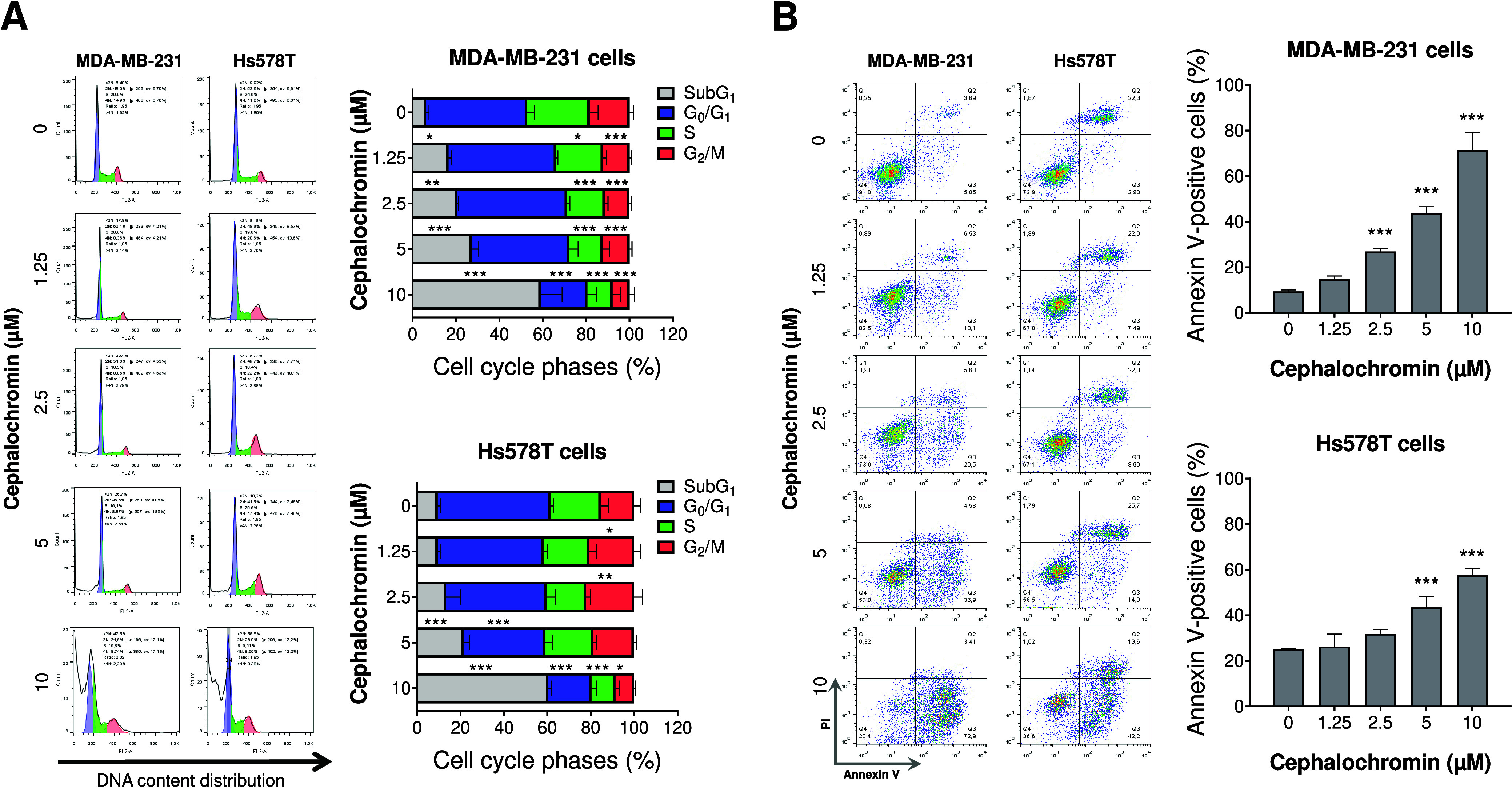
**Cephalochromin disrupts cell cycle progression and induces apoptosis in breast cancer models.** (A) DNA content distribution was analyzed by propidium iodide staining and flow cytometry in MDA-MB-231 and Hs578T cells treated with vehicle or cephalochromin (1.25, 2.5, 5, and 10 μM) for 48 h. Representative histograms for each condition are shown. The bar graph represents the mean ± SD from at least three independent experiments. Statistical significance is indicated; *p < 0.05, **p < 0.01, ***p < 0.001; ANOVA followed by Bonferroni post hoc test. (B) MDA-MB-231 and Hs578T cells were stained with APC-annexin V and propidium iodide (PI) following treatment with vehicle or cephalochromin (1.25, 2.5, 5, and 10 μM) for 48 h. Representative dot plots are shown for each condition. The apoptotic cell population (annexin V+ cells) is represented by the upper and lower right quadrants (Q2 + Q3). Bar graphs show the mean ± SD from at least three independent experiments. Statistical significance and cell line information are indicated in the graphs; ***p < 0.0001; ANOVA followed by Bonferroni post hoc test.

### Cephalochromin Suppresses Survivin Expression and Generates a Tumor-Suppressive Molecular Network in Breast Cancer Cells

Next, we evaluated molecular markers previously reported to be modulated by cephalochromin^17^ and conducted an exploratory analysis of key genes involved in cell cycle progression, apoptosis, DNA damage, and autophagy. In MDA-MB-231 and Hs578T cells, cephalochromin treatment increased PARP1 cleavage (a marker of apoptosis) and γH2AX expression (a marker of DNA damage), reduced survivin/BIRC5 expression (an inhibitor of apoptosis, IAP), and promoted the accumulation of SQSTM1/p62 and LC3B–II (markers of autophagy; Figure 3A).

**Figure 3:**
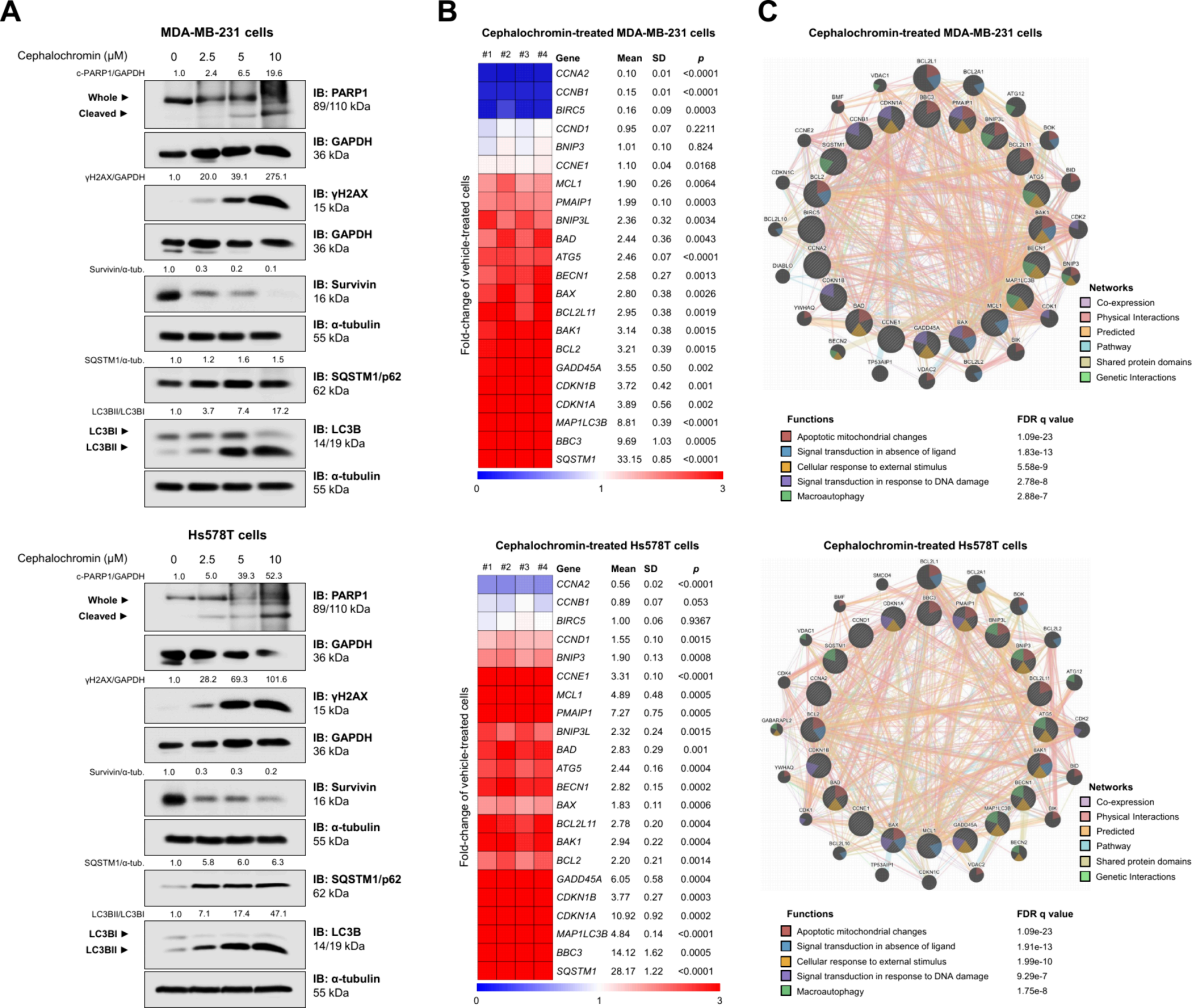
**Cephalochromin downregulates survivin/BIRC5 and induces a tumor-suppressive molecular profile in breast cancer cells.** (A) Western blot analysis was performed to assess the expression of PARP1, γH2AX, survivin/BIRC5, SQSTM1/p62, and LC3B in whole-cell extracts from MDA-MB-231 and Hs578T cells. Cells were treated with either vehicle or cephalochromin (2.5, 5, and 10 μM) for 48 h, as indicated. Membranes were reprobed with GAPDH or α-tubulin antibodies as the loading control. Relative protein expression was quantified by densitometric analysis and is presented in the figure. (B) The heatmap illustrates the gene expression profile of MDA-MB-231 and Hs578T cells treated with either vehicle or cephalochromin (5 μM) for 48 h. Blue indicates decreased mRNA levels, while red indicates increased mRNA levels, normalized to vehicle-treated controls (*n* = 4). Fold-change (FC), standard deviation (SD), and *p*-values were calculated using Student’s *t* test. A gene network of cephalochromin-modulated genes was generated using the GeneMANIA database (https://genemania.org/). Genes with significant modulation are represented as crosshatched circles, while interacting genes added by the software are shown as noncrosshatched circles. The biological interactions, associated functions, and false discovery rate (FDR) q-values are detailed in the figure.

The exploratory analysis revealed strong modulation of the evaluated genes, with 20 of 22 genes showing significant expression changes following cephalochromin exposure in both cell models (Figure 3B). Molecular network analysis linked these gene modulations to apoptotic mitochondrial changes, signal transduction in the absence of ligand, cellular responses to external stimuli, DNA damage response signaling, and macroautophagy (Figure 3C), corroborating findings from previous cellular and protein expression assays. Interestingly, in MDA-MB-231 cells, but not in Hs578T cells, survivin/BIRC5 expression was modulated at the mRNA level, suggesting that cephalochromin may regulate survivin not only by suppressing transcription but also by influencing protein stability and degradation.

### Cephalochromin Enhances the Efficacy of Clinically Used Chemotherapeutic Agents in Breast Cancer Cells

Finally, given that cephalochromin demonstrated significant antineoplastic effects as a monotherapy in *in vitro* breast cancer models, we investigated its effects in combination with clinically used chemotherapeutic agents, specifically, paclitaxel and doxorubicin. These compounds alone were also included as references in the combination experiments. In MDA-MB-231 and Hs578T cells, cephalochromin treatment exhibited additive effects in enhancing paclitaxel-induced viability reduction (ZIP score >5). Notably, the combination of cephalochromin with doxorubicin demonstrated synergistic effects in both breast cancer models evaluated (ZIP score >10) (Figure 4). Together, these findings suggest that cephalochromin may enhance tumor cell sensitivity to standard therapeutic regimens used in breast cancer treatment.

**Figure 4:**
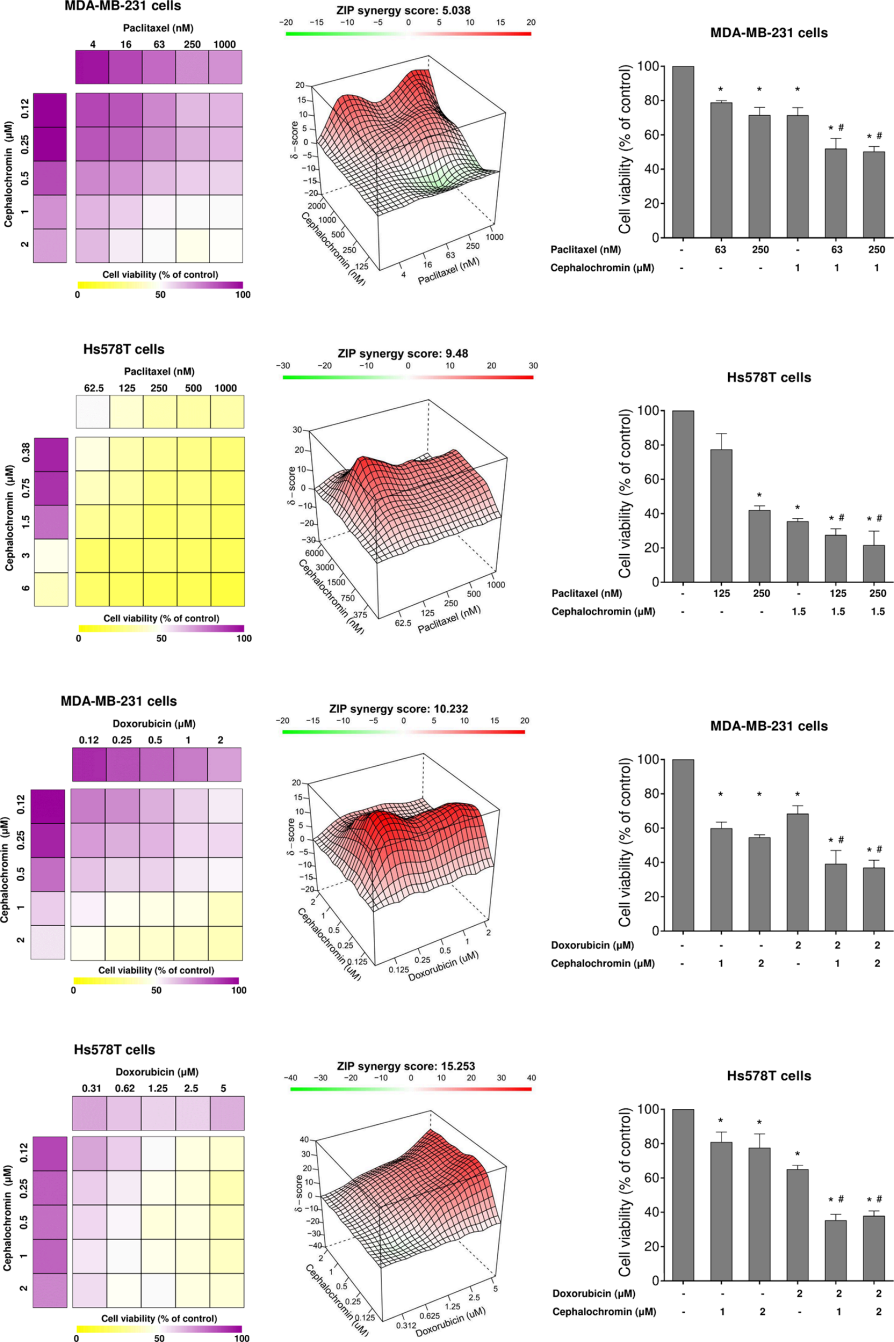
**Cephalochromin potentiates the antineoplastic effects of clinically used chemotherapeutic agents in breast cancer cells.** Dose–response cytotoxicity assays for cephalochromin combined with paclitaxel and cephalochromin combined with doxorubicin were conducted using the methylthiazolethrazolium (MTT) assay in MDA-MB-231 and Hs578T cells. Breast cancer cells were exposed to vehicle or graded concentrations of cephalochromin, paclitaxel, or doxorubicin, either alone or in combination, for 72 h, as indicated. Values are expressed as the percentage of vehicle-treated cells. The ZIP synergy score was calculated using SynergyFinder software (https://synergyfinder.fimm.fi/). Bar graphs highlight the context-relevant combinations. *p < 0.05 treatment versus vehicle and ^#^ *p* < 0.05 monotherapy versus combination therapy; ANOVA with Bonferroni post-test. Results represent the mean of at least three independent experiments.

In this study, we characterized the antineoplastic effects of cephalochromin in breast cancer cell models including reduced cell viability and clonal growth, delayed cell cycle progression, disrupted autophagy, and induced apoptosis and DNA damage. The selection of breast cancer models for this study was based on the potential target of cephalochromin, the survivin protein, which plays a crucial role in the disease phenotype and is considered an important diagnostic and prognostic marker in meta-analysis studies.^13,14^ The potential antineoplastic effects of cephalochromin were previously investigated in another study, initially testing a panel of tumor cell lines including A549 (lung cancer), Hep3B (liver cancer), Caco-2 (colon cancer), HT1080 (sarcoma), Huh7 (liver cancer), and SW1353 (chondrosarcoma), with functional and molecular studies being further explored only in the lung cancer model.^17^ In that study, the IC_50_ values for cephalochromin ranged from 2.72 to 14.31 μM across the tested cancer models. In contrast, for all TNBC models analyzed in the present study, the IC_50_ values were lower than the previously observed lower limit (0.6 to 1.6 μM),^17^ suggesting that TNBC models are highly sensitive to the drug.

Regarding cellular phenotype, the accumulation of Hs578T breast cancer cells in the G_2_/M phase differs from the initial findings in A549 lung cancer cells, in which cell accumulation occurred in the G_0_/G_1_ phase.^17^ Survivin is regulated during the G_2_/M phase of the cell cycle and is essential for proper chromosome segregation during cell division. As a component of the chromosomal passenger complex (CPC), survivin facilitates CPC function by directing the complex to specific sites on the mitotic spindle, where it regulates chromosome segregation and cytokinesis.^18–21^ The observed reduction in cyclin A (*CCNA2*) and cyclin B (*CCNB1*) expression, along with the increased levels of cyclin-dependent kinase inhibitors *CDKN1A* (p21) and *CDKN1B* (p27), corroborates the cell cycle arrest at the G_2_/M phase.^22,23^ Conversely, the accumulation of cells in the subG_1_ phase following cephalochromin exposure in both analyzed breast cancer cell lines may be associated with the inhibition of survivin’s canonical function as an inhibitor of apoptosis (IAP),^7,24^ contributing to apoptotic induction. Indeed, MDA-MB-231 cells, which exhibited the highest level of subG_1_ accumulation, were also the most sensitive to apoptosis, as determined by phosphatidylserine externalization. Collectively, our findings reinforce the on-target effects of cephalochromin on survivin in breast cancer models.

Among the key molecular markers, we highlight DNA damage and autophagy markers triggered by cephalochromin treatment in breast cancer cells. The increased phosphorylation of H2AX at serine 139 (γH2AX) is a widely accepted marker of DNA damage.^25^ Additionally, several DNA damage response genes, including *CDKN1A*, *CDKN1B*, *GADD45A*, *BAX*, *PMAIP1*, and *BBC3*, exhibited increased expression following drug exposure.^26–29^ Regarding autophagy, our study provides a more comprehensive molecular characterization than previously reported. In lung cancer cells, cephalochromin-induced autophagy was identified based on increased LC3BII expression.^17^ Our findings confirm this autophagy induction, as evidenced by the upregulation of LC3BII and autophagy-related genes such as *ATG5*, *BECN1* (beclin 1), *MAP1LC3B* (LC3B), and *SQSTM1* (p62). Simultaneously, we observed an accumulation of SQSTM1/p62, indicating a blockage in the late stages of the autophagic flux, suggesting a state of autophagic catastrophe, which may contribute to apoptosis.^30^

A novel and relevant aspect of this study is that cephalochromin enhances the efficacy of clinically used chemotherapeutic agents. In the context of breast cancer, paclitaxel and doxorubicin are considered cornerstone chemotherapeutic agents.^31^ Although both TNBC cell lines demonstrated sensitivity to paclitaxel and doxorubicin, it is important to note that Hs578T and MDA-MB-231 cells exhibit distinct molecular characteristics that may influence their response to chemotherapy.^32^ For instance, MDA-MB-231 cells are highly invasive and metastatic, which may confer greater chemoresistance compared to that of less aggressive cell lines. This resistance is likely associated with the activation of survival signaling pathways, such as the PI3K/AKT/mTOR pathway.^33^ Notably, paclitaxel treatment can increase survivin expression, which has been linked to evasion of taxol-induced apoptosis.^34^ Furthermore, previous studies have shown that survivin silencing via siRNA enhances paclitaxel sensitivity.^35,36^ Similarly, survivin overexpression promotes doxorubicin resistance, and pretreatment with survivin-targeting siRNA enhances doxorubicin-induced cytotoxicity.^37,38^ Based on our findings, cephalochromin represents a promising pharmacological alternative for suppressing survivin expression and acting as a chemosensitizer in breast cancer treatment. Additionally, it may overcome limitations associated with siRNA use, such as stability and the unpredictable pharmacokinetics and pharmacodynamics that hinder systemic clinical application.^39^

Despite providing insights into cephalochromin’s mechanism of action in breast cancer, this study has limitations. Experiments were performed only in cell line models, and future studies using *in vivo* systems are necessary to assess efficacy and toxicity and strengthen the evidence. Additionally, studies of apoptosis and autophagy would benefit from the concomitant use of specific inhibitors (e.g., Z-VAD-FMK for apoptosis; 3-MA, chloroquine, or bafilomycin A1 for autophagy). Our results support previous evidence identifying survivin as a relevant target, but multiple genes were modulated by the drug. Cephalochromin’s multitarget effects may be advantageous in oncology, as they can reduce the likelihood of resistance.^40,41^ Future studies are needed to map molecular targets and validate these findings in animal models. Preliminary findings from spheroid-based breast cancer models suggest that drug efficacy may be compromised (Figure S2), which warrants further investigation. Drug penetration in tridimensional models is often impaired^42^ and may require optimization through advanced formulations or nanotechnology, as demonstrated in studies where survivin-targeting siRNA was coencapsulated with paclitaxel or doxorubicin.^36,38^ Nevertheless, our study expands the current knowledge of cephalochromin as a potential antineoplastic drug.

## Conclusion

In summary, our findings demonstrate that cephalochromin exhibits significant antineoplastic potential in breast cancer models by reducing cell viability, inhibiting clonal growth, and inducing DNA damage, apoptosis, and cell cycle arrest. Furthermore, its ability to suppress survivin expression and enhance the effects of clinically relevant chemotherapeutic agents underscores its potential as a therapeutic candidate. However, further studies are needed to validate its efficacy and safety. Overall, this work broadens our understanding of cephalochromin and opens new avenues for its application in breast cancer treatment.

## Experimental Section

### General Experimental Procedures

HPLC-grade solvents from Merck were used for the HPLC chromatography analyzes. Analytical HPLC analyses were carried out on an Agilent 1260 system (1260 Infinity LC system, Agilent Technologies, La Jolla, CA, USA) equipped with an ultraviolet spectrum scanning detector by arrangement of photodiodes with a 60 mm flow cell. A Zorbax Eclipse plus reverse phase C_18_ column (4.6 × 150 mm, 3.5 μm, Agilent, La Jolla, CA, USA) was used as the stationary phase, a flow rate of 1.0 mL/min was employed for analysis on an analytical scale, and the column temperature was 45 °C. The injection volume of the sample was 3 μL, and the sample was dissolved in methanol at a concentration of 1 mg/mL. For the separation of compounds, the Agilent 1200 semipreparative chromatography system (1200 LC system, Agilent Technologies, La Jolla, CA, USA) was used with a C_18_ Zorbax eclipse plus LC-18 column (25 cm × 10 mm, 5 μm, Agilent, La Jolla, CA, USA), a flow rate of 4.2 mL·min^–1^ for solvents, and a column temperature of 45 °C. The injection volume of the sample was 200 μL, and the sample was dissolved in methanol at a concentration of 100 g/L. Both scales (analytical and semipreparative) were employed as the solvents: solvent A was milli-Q water acidified with 0.1% acetic acid (v/v), and solvent B was acetonitrile (ACN).

Nuclear magnetic resonance (NMR) spectra were recorded on a Bruker Avance III 500 MHz spectrometer (Bruker, Bremen, Germany) equipped with a 5 mm probe, operating at 500.11 MHz for ^1^H NMR and 125.5 MHz for ^13^C NMR, at IQ-USP. Chloroform-D from Sigma-Aldrich was used as the solvent, and all chemical shifts referred to internal TMS. All NMR spectra were processed using Mestrelab MestreNOVA software (Figure S4).

### Fungal Growth, Extraction, Isolation, and Identification of Compound

Endophytic fungus was recovered from leaves of *Moquiniastrum polymorphum* (Less.) G. Sancho (Asteraceae; voucher specimen Farinella 02), and according to molecular data of the ITS region was recognized as *Alternaria* sp.^43^ The strain was cultured on potato dextrose agar (PDA) at 25 °C for 60 days using 150 Petri dishes and, subsequently, extracted with ethyl acetate. The crude extract (1.53 g) was defatted with hexane and fractionated by semipreparative HPLC with H_3_O^+^–ACN gradient (10–100% ACN) for 30 min. The isolated compound was eluted at 20 min and identified as cephalochromin through NMR data and comparison with literature data.^44^

#### Cephalochromin (Figure 1A)

Yellow-greenwish, amorphous powder. ^1^H NMR (CDCl_3_): δ_H_ 15.07 (s, 2H, 5-OH, 5′-OH), 9.68 (s, 2H, 6-OH, 6′-OH), 6.53 (s, 2H, H-7, H-7′), 5.94 (s, 2H, H-10, H-10′), 4.49 (m, 2H, H-2, H-2′), 2.71 (d, *J* = 3.1 Hz, 2H, H-3, H-3′), 2.69 (s, 2H, H-3′), 1.42 (d, *J* = 6.3 Hz, 6H, H-11, H-11′); ^13^C NMR (CDCl_3_): δ 198.4 (C-4, C-4′), 164.6 (C-5, C-5′), 160.9 (C-6, C-6′), 160.1 (C-8, C-8′), 156.4 (C-10a, C-10a′), 142.1 (C-9a, C-9a′), 105.5 (C-5a, C-5a’), 102.5 (C-4a, C-4a′), 102.2 (C-9, C-9′), 100.0 (C-7, C-7′), 99.6 (C-10, C-10′), 73.3 (C-2, C-2′), 43.2 (C-3, C-3′), 20.9 (C-11, C-11′).

### Cell Culture and Chemical Reagents

The MCF-10A, MDA-MB-231, BT-549, HCC70, and HCC1937 cell lines were kindly provided by Dr. Silvina Odete Bustos (Instituto do Cancer do Estado de São Paulo Octavio Frias de Oliveira, São Paulo, Brazil), while the MCF-12A, MCF-7, T47D, and Hs578T cell lines were obtained from Prof. Glaucia Maria Machado Santelli (Institute of Biomedical Sciences, São Paulo, Brazil). Breast cancer cells were cultured in the appropriate medium supplemented with 10% fetal bovine serum (FBS) according to the guidelines of the American Type Culture Collection (ATCC) or Deutsche Sammlung von Mikroorganismen and Zellkulturen (DSMZ). Nonmalignant human breast epithelial cell lines, MCF-10A and MCF-12A, were cultured in phenol red-free Dulbecco’s Modified Eagle’s Medium: Nutrient Mixture F-12 (DMEM/F-12) (Sigma-Aldrich, St. Louis, MO, USA), supplemented with 5% horse serum, 0.02 μg/mL EGF, 0.5 μg/mL hydrocortisone, 0.1 μg/mL cholera toxin, and 10 μg/mL insulin. The cultures also contained 1% penicillin/streptomycin and were maintained at 37 °C in a humidified atmosphere with 5% CO_2_. Cultures were routinely tested and confirmed to be mycoplasma-free during the experiments. Cephalochromin was diluted in DMSO at a concentration of 10 mM. Doxorubicin and paclitaxel were purchased from Sigma-Aldrich (St. Louis, MO, USA) and were diluted and stored according to the manufacturer’s instructions.

### Cell Viability

Cell viability was assessed using the MTT assay following the manufacturer’s protocol (Sigma-Aldrich, St. Louis, MO, USA). In brief, 5 × 10^3^ cells per well were plated in 96-well plates and treated with increasing concentrations of the tested compounds in the appropriate culture medium. After 24, 48, and/or 72 h of incubation, 10 μL of MTT solution (5 mg/mL) was added to each well and incubated for 4 h at 37 °C. The reaction was terminated by adding 100 μL of 0.1 N HCl in isopropanol, and absorbance was measured at 570 nm using an automated plate reader. Each condition was tested in at least three replicates, and experiments were independently repeated at least three times. Inhibitory concentration of 50% (IC_50_) values were determined using nonlinear regression analysis in GraphPad Prism 8 software (GraphPad Software, Inc., San Diego, CA, USA). The selectivity index (SI) was calculated as the ratio between the IC_50_ determined in nonmalignant cells and the IC_50_ in cancer cells. For combination studies, MDA-MB-231 and Hs578T cells were treated with graded doses of cephalochromin, doxorubicin, and paclitaxel, either alone or in combination, for 72 h. Data visualization was performed using the Multiple Experiment Viewer (MeV) 4.9.0 software,^45^ and the ZIP synergy score was calculated using SynergyFinder (https://synergyfinder.fimm.fi/).

For spheroid studies, breast cancer cell lines were seeded at a density of 1 × 10^4^ cells in 100 μL per well in a 96-well plate, with 65 μL of 1% agarose previously added to the bottom of each well. The plates were incubated for 4 days at 37 °C under a 5% CO_2_ atmosphere until spheroids were formed. Subsequently, the spheroids were treated with the vehicle or cephalochromin for 72 h. Images were captured using an EVOS AME-3302 inverted digital microscope. Spheroid viability was assessed by quantifying ATP levels using the CellTiter-Glo 3D Cell Viability Kit (Promega, Madison, WI, USA), following the manufacturer’s protocol.

### Colony Formation

Breast cancer cell lines (1 × 10^3^ cells per 35 mm dish) were treated with either the vehicle or increasing concentrations of cephalochromin (0.5, 1, 5, 10, and 20 μM). Following 10–14 days of incubation, colonies were stained with 0.5% crystal violet (Sigma-Aldrich) in 10% ethanol. Images were acquired using the G:BOX Chemi XX6 gel document system (Syngene, Cambridge, UK) and analyzed with ImageJ software (US National Institutes of Health, Bethesda, MD, USA).

### Cell Cycle Analysis

A total of 1 × 10^5^ cells were plated per well in 60 mm dishes and treated with either the vehicle or cephalochromin (1.25, 2.5, 5, and 10 μM). After 48 h, cells were collected, fixed in 70% ethanol, and stored at 4 °C for a minimum of 2 h before analysis. Fixed cells were then stained with 20 μg/mL propidium iodide (PI) and 10 μg/mL RNase A for 30 min at room temperature in a light-protected environment. DNA content distribution was assessed using a FACSCalibur flow cytometer (Becton Dickinson, Franklin Lakes, NJ, USA) and analyzed with FlowJo software (Treestar, Inc., San Carlos, CA, USA).

### Apoptosis Analysis

MDA-MB-231 and Hs578T cells (1 × 10^5^ per well) were plated in 12-well plates and treated with either the vehicle or cephalochromin (1.25, 2.5, 5, and 10 μM) for 48 h. After treatment, cells were washed with ice-cold phosphate-buffered saline (PBS) and resuspended in a binding buffer containing 1 μg/mL propidium iodide (PI) and 1 μg/mL APC-labeled annexin V. Following a 15 min incubation at room temperature in a light-protected environment, samples were analyzed by flow cytometry using a FACSCalibur system (Becton Dickinson, Franklin Lakes, NJ, USA). A total of 10,000 events were recorded per sample.

### Western Blot

Total protein was extracted using a lysis buffer containing 100 mM Tris (pH 7.6), 1% Triton X-100, 2 mM PMSF, 10 mM Na_3_VO_4_, 100 mM NaF, 10 mM Na_4_P_2_O_7_, and 10 mM EDTA. Protein samples (30 μg) were separated by SDS-PAGE and transferred onto nitrocellulose membranes. After blocking with 5% nonfat milk, membranes were incubated with specific primary antibodies diluted in blocking buffer, followed by HRP-conjugated secondary antibodies. Western blot detection was performed using the SuperSignal West Dura Extended Duration Substrate System (Thermo Fisher Scientific) and imaged with the G:BOX Chemi XX6 gel document system (Syngene, Cambridge, UK (Syngene). The following antibodies were used: PARP1 (no. 9542), γH2AX (no. 9718), survivin (no. 2808), SQSTM1/p62 (no. 88588), LC3B (no. 2775), GAPDH (no. 5174), and α-tubulin (no. 2144) were obtained from Cell Signaling Technology (Danvers, MA, USA). Band intensities were quantified using UN-SCAN-IT gel 6.1 software (Silk Scientific, Orem, UT, USA). Protein content was normalized to that of GAPDH or α-tubulin.

### Quantitative PCR

Total RNA was extracted using TRIzol reagent (Thermo Fisher Scientific, San Jose, CA, USA). Complementary DNA (cDNA) was synthesized from 1 μg of RNA with the High-Capacity cDNA Reverse Transcription Kit (Thermo Fisher Scientific). Quantitative PCR (qPCR) was performed on a QuantStudio 3 Real-Time PCR System using the SybrGreen System and specific primers (Table S2). *HPRT1* and *ACTB* were used as reference genes, and relative gene expression was calculated using the 2^–ΔΔCT^ method.^46^ A no-template control was included for each primer pair to verify the absence of contamination. To assess the specificity, a dissociation curve analysis was conducted at the end of each run. Data visualization was carried out using MeV 4.9.0 software.^45^ Gene networks highlighting modulated genes and key biological interactions were generated using the GeneMANIA database (https://genemania.org/).

### Statistical Analysis

Statistical analyses were performed using GraphPad Instat 8 (GraphPad Software, Inc.). Group comparisons were conducted using ordinary one-way ANOVA followed by Bonferroni’s multiple comparison test or Student’s *t* test. A *p*-value of <0.05 was considered statistically significant.

## Supplementary Material



## Data Availability

The data generated or analyzed in this study during the current study are available from the corresponding author on reasonable request.

## Notes

Additional experimental details, materials, and methods; dose–response curves of doxorubicin and paclitaxel in breast cancer cell lines; effects of cephalochromin on breast cancer cell spheroids; survivin expression in malignant and nonmalignant breast cell lines; NMR spectra of cephalochromin; selectivity index data; and primer sequences and concentrations used for quantitative PCR (PDF)
