# Supplementary material for: Cephalochromin Effects in Triple-Negative Breast Cancer Cells: Apoptosis Induction and Modulation of Survival Pathways
Source: J Nat Prod. 2025 Dec 4;88(12):2851–9. doi: 10.1021/acs.jnatprod.5c01020 (PMC12751111; doi:10.1021/acs.jnatprod.5c01020)
Supplement: Supplementary file 1 [file np5c01020_si_001.pdf]

Supporting Information

Cephalochromin Effects in Triple-Negative Breast Cancer Cells: Apoptosis Induction and Modulation of Survival Pathways

Isabelle Diccini [1], Natália Sudan Parducci [1], Bruna Oliveira de Almeida [1], Victor Farinella [2], Patrick Castilho dos Santos [2], Livia Bassani Lins de Miranda [1], Sabrina Mendes Botelho [3], Keli Lima [1,4], Jorge Antonio Elias Godoy Carlos[1], Anali Del Milagro Bernabe Garnique[1], Marcelo José Pena Ferreira [2], Leticia Veras Costa-Lotufo [1,*], João Agostinho Machado-Neto [1,*]

[1] Department of Pharmacology, Institute of Biomedical Sciences, University of São Paulo, São Paulo, CEP 05508-900, Brazil

[2] Department of Botany, Institute of Biosciences, University of São Paulo, São Paulo, CEP 05508-090, Brazil

[3] São Carlos Institute of Chemistry, University of São Paulo, São Carlos, CEP 13563-120, Brazil

[4] Department of Internal Medicine, Faculty of Medicine, University of São Paulo, São Paulo, CEP 05403-000, Brazil

*costalotufo@usp.br; jamachadoneto@usp.br

Table of contents

**Figure S1. Dose-dependent cytotoxicity of doxorubicin and paclitaxel in MDA-MB-231 and Hs578T cells.**	**S-2**
**Figure S2. Assessment of the impact of cephalochromin treatment on breast cancer cell spheroids.**	**S-3**
**Figure S3. Survivin expression in non-malignant and malignant breast cell lines.**	**S-4**
**Figure S4. Nuclear magnetic resonance spectra of cephalochromin.**	**S-5**
**Table S1. Selectivity index of cephalochromin in breast cancer cells.**	**S-6**
**Table S2. Primer sequences and concentrations for quantitative PCR.**	**S-7**

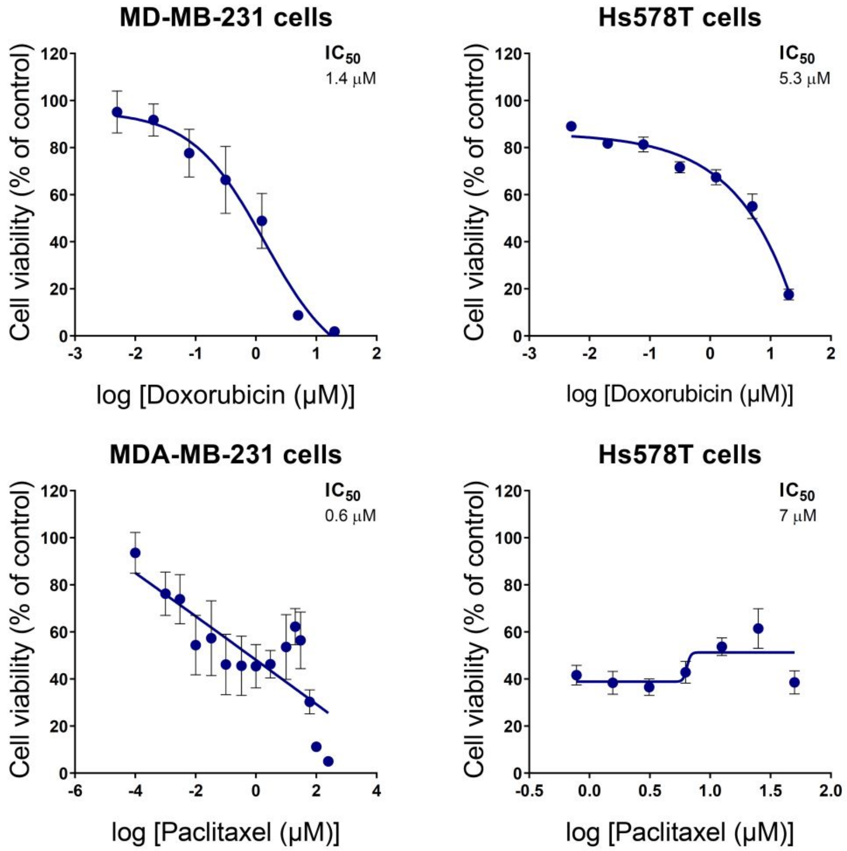

Figure S1. Dose-dependent cytotoxicity of doxorubicin and paclitaxel in MDA-MB-231 and Hs578T cells. Dose-dependent cytotoxicity was evaluated using the methylthiazolyl tetrazolium (MTT) assay in MDA-MB-231 and Hs578T, BT-549 cells treated with either vehicle or increasing concentrations of doxorubicin and paclitaxel for 72 hours.

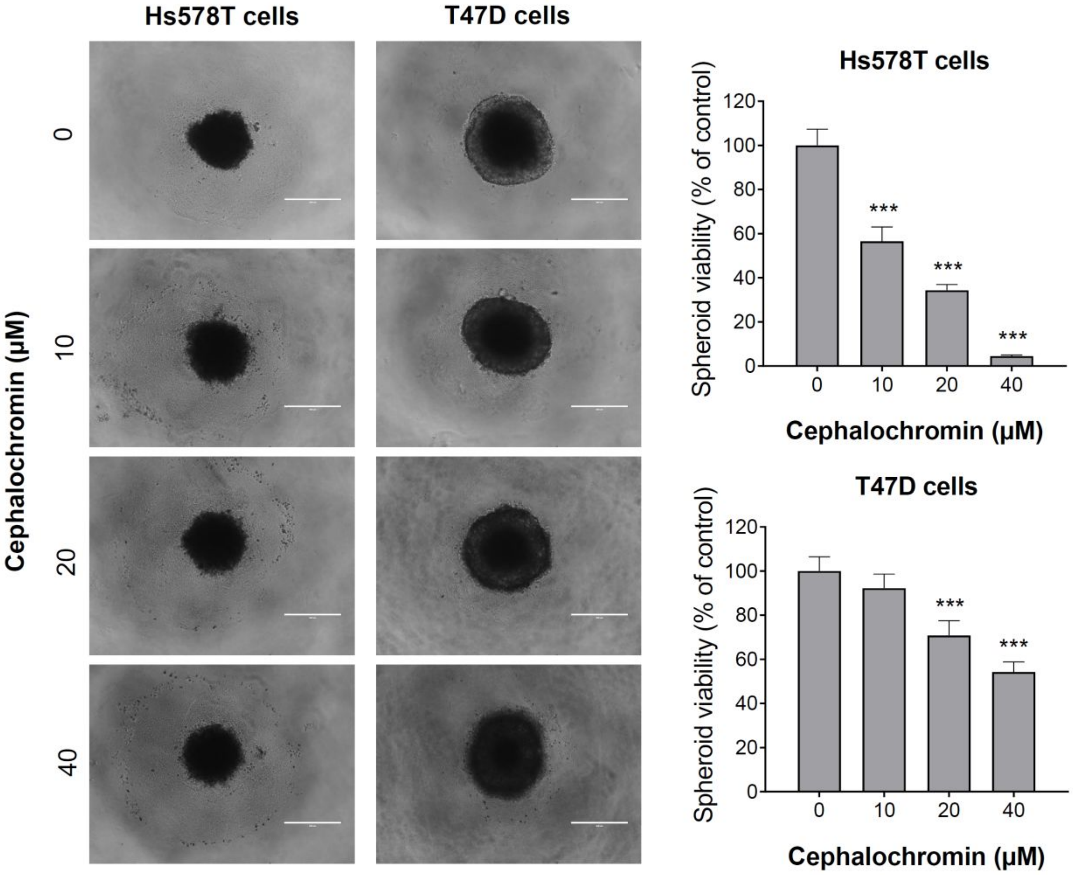

Figure S2. Assessment of the impact of cephalochromin treatment on breast cancer cell spheroids. The Hs578T and T47D cell lines were seeded at a density of 1 × 10^4 cells in 100 µL per well in 96-well plates with 65 µL of 1% agarose previously added to the bottom of each well. The plates were incubated for 4 days at 37°C in a 5% CO_2 atmosphere until spheroids were formed. Subsequently, the spheroids were treated with the vehicle or cephalochromin (10, 20, and 40 µM) for 72 hours. Bar graphs show the mean ± standard deviation (SD) of at least three independent experiments. ***$p < 0.05$; ANOVA followed by Bonferroni post-hoc test**.**

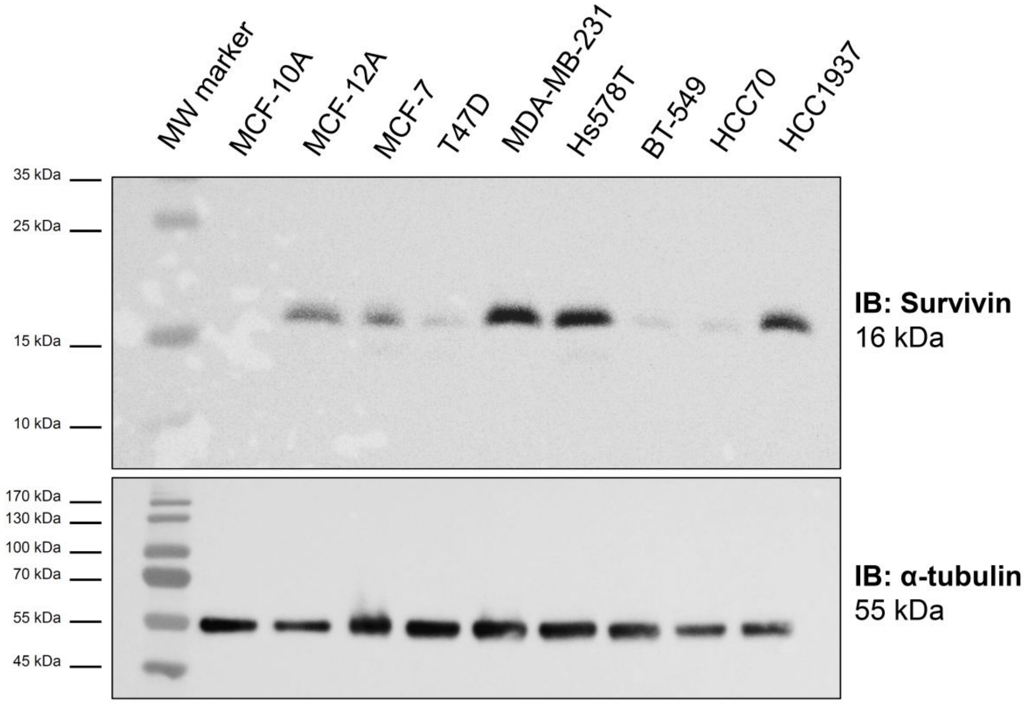

Figure S3. Survivin expression in non-malignant and malignant breast cell lines. Western blot analysis was performed to assess the expression of survivin/BIRC5 in whole-cell extracts from non-malignant mammary epithelial cells (MCF-10A and MCF-12A) and breast cancer cell lines (MCF-7, T47D, MDA-MB-231, Hs578T, BT-549, HCC70, and HCC1937). Membranes were probed with α-tubulin antibody as loading control.

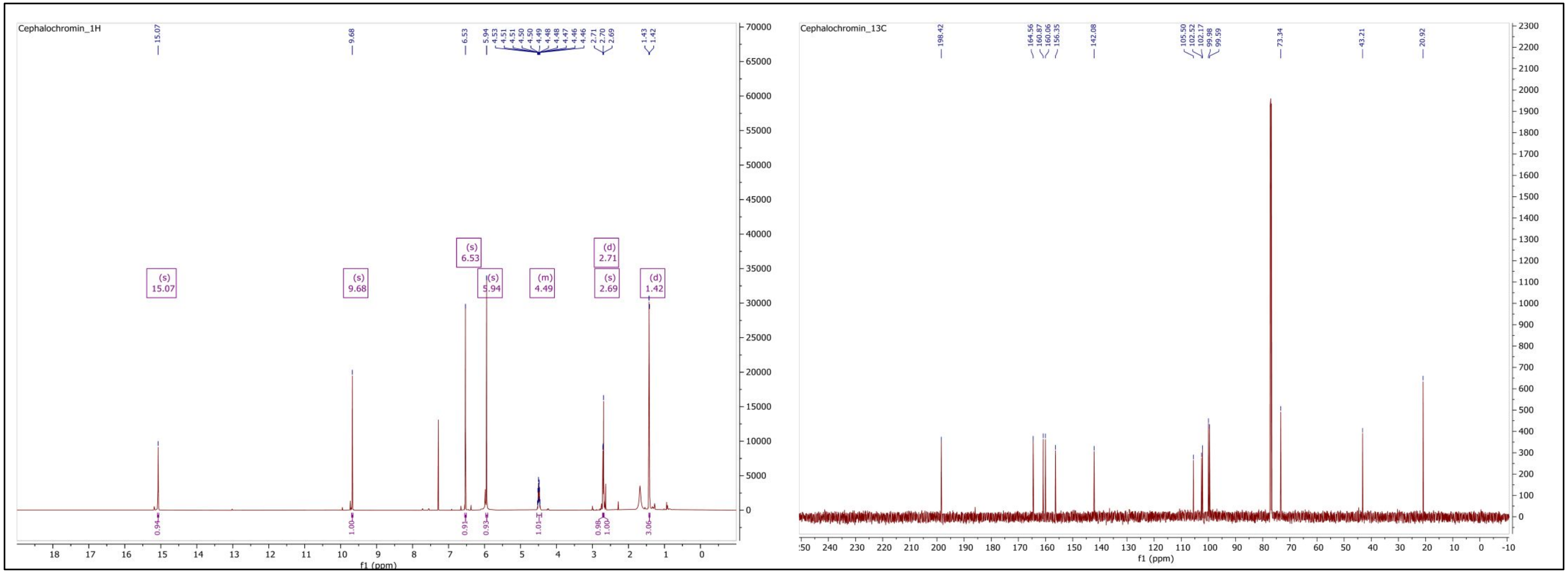

Figure S4. Nuclear magnetic resonance spectra of cephalochromin.

Table S1. Selectivity index of cephalochromin in breast cancer cells.

Cell line	Characteristics	SI (MCF-10A)	SI (MCF-12A)
MCF-10A	Non-malignant	1.0 (ref.)	-
MCF-12A	Non-malignant	-	1.0 (ref.)
MCF-7	ER^+, PR^+, $HER2^-$	0.26	0.14
T47D	ER^+, PR^+, $HER2^-$	0.24	0.13
MDA-MB-321	ER^-, PR^-, $HER2^-$	5.33	2.83
Hs578T	ER^-, PR^-, $HER2^-$	3.55	1.89
BT-549	ER^-, PR^-, $HER2^-$	2.91	1.55
HCC70	ER^-, PR^-, $HER2^-$	3.20	1.70
HCC1937	ER^-, PR^-, $HER2^-$	2.00	1.06

Abbreviations: SI, selectivity index; ER, estrogen receptor; PR, progesterone receptor; HER2, human epidermal growth factor receptor 2.

Table S2. Primer sequences and concentrations for quantitative PCR.

Gene[1]	Sequence	Concentration
ACTB	FW: AGGCCAACCGCGAGAAG<br>RV: ACAGCCTGGATAGCAACGTACA	150 nM
ATG5	FW: GGGCCATCAATCGGAAAC<br>RV: AGCCACAGGACGAAACAG	300 nM
BAD	FW: CACCAGCAGGAGCAGCCAAC<br>RV: CGACTCCGGATCTCCACAGC	300 nM
BAK1	FW: TGAGTACTTCACCAAGATTGCCA<br>RV: AGTCAGGCCATGCTGGTAGAC	300 nM
BAX	FW: GAGCTGCAGAGGATGATTGC<br>RV: CAGCTGCCACTCGGAAAA	300 nM
BBC3	FW: GACCTCAACGCACAGTACGAG<br>RV: AGGAGTCCCATGATGAGATTGT	300 nM
BCL2	FW: ATGTGTGTGGAGAGCGTCAA<br>RV: ACAGTTCCACAAAGGCATCC	300 nM
BCL2L11	FW: ATGTCTGACTCTGACTCTCG<br>RV: CCTTGTGGCTCTGTCTGTAG	300 nM
BECN1	FW: TCTGAAGAGGACCTGGACCCT<br>RV: GGCTCACGTCCATCTCGTC	300 nM
BIRC5	FW: GCCCAGTCTTTCTTCTGCTTCA<br>RV: GACCTTTCTCCGCAGTTTCCTC	300 nM
BNIP3	FW: ATATGGGATTGGTCAAGTCGG<br>RV: CGCTCGTGTTCCTCATGCT	300 nM
BNIP3L	FW: ACACCAGCAGGGACCATAGC<br>RV: TTTCTTCAAAGCCTCGACTTCC	300 nM
CCNA2	FW: GCCTTTCATTTAGCACTCTACA<br>RV: CAGGGTATATCCAGTCTTTCG	300 nM
CCNB1	FW: GTCTCCATTATTGATCGGTTCATG<br>RV: CCAATTTCTGGAGGGTACATTTCT	300 nM
CCND1	FW: CTCGGTGTCCTACTTCAAATG<br>RV: AGCGGTCCAGGTAGTTCAT	300 nM
CCNE1	FW: TATATGGCGACACAAGAAAATG<br>RV: GTGCAACTTTGGAGGATAGA	300 nM
CDKN1A	FW: TGTCACTGTCTTGTACCCTTGT<br>RV: GCCGGCGTTTGGAGTGGTAG	300 nM
CDKN1B	FW: ACTCTGAGGACACGCATTTGGT<br>RV: TCTGTTCTGTTGGCTCTTTTGTT	300 nM
GADD45A	FW: AAGGATGGATAAGGTGGGG<br>RV: CTGGATCAGGGTGAAGTGG	300 nM
HPRT1	FW: GAACGTCTTGCTCGAGATGTGA<br>RV: TCCAGCAGGTCAGCAAAGAAT	150 nM
MAP1LC3B	FW: AAGGCGCTTACAGCTCAATG<br>RV: CTGGGAGGCATAGACCATGT	300 nM
MCL1	FW: GTAATAACACCAGTACGGACGG<br>RV: TCCCGAAGGTACCGAGAGAT	300 nM
PMAIP1	FW: CGCGCAAGAACGCTCAACC<br>RV: CACACTCGACTTCCAGCTCTGCT	300 nM
SQSTM1	FW: TGAGGAACAGATGGAGTCGGATAA<br>RV: GGGACTGGAGTTCACCTGTAGACG	300 nM

[1]Genes are reported according to the Human Genome Organisation (HUGO) Gene Nomenclature Committee (HGNC).

Abbreviations: FW, forward; RV, reverse.
